# Supplementary material for: Flushing peripheral intravenous catheters: A scoping review
Source: PLoS One. 2025 Aug 19;20(8):e0330125. doi: 10.1371/journal.pone.0330125 (PMC12364367; doi:10.1371/journal.pone.0330125)
Supplement: S1 Table — (DOCX) [file pone.0330125.s001.docx]

Appendix I: Search terms and synonyms

| Concept 1 (PVC) | Concept 2 (flushing method) |
| --- | --- |
| peripheral venous catheter*  peripheral intravenous catheter*  PIVC*  PVC*  Short peripheral catheter*  SPC*  Midline catheter*  MC*  peripheral intravenous cannula*  peripheral venous cannula*  vascular access device*  VAD*  venous catheter*  intravenous access device*  IVAD*  intravenous catheter*  IVC  intravenous cannula*  IV catheter*  IV cannula*  venous cannula* | flush* |

Appendix Ⅱ The data extraction instrument

| **The basic information about citations** | **Study design of citations** | **Content of the citation** |
| --- | --- | --- |
| author(s) | study design | intravenous catheter type |
| year of publication | aim/purpose | flushing techniques |
| country or origin of the publication | population and sample size | Flushing methods  flushing speed  flushing volume  flushing frequency  flushing interval |
| journal | trail intervention (type, duration, comparator) |  |
|  | outcome assessment |  |
|  | clinical trial registration |  |
