# Supplementary material for: Flushing peripheral intravenous catheters: A scoping review
Source: PLoS One. 2025 Aug 19;20(8):e0330125. doi: 10.1371/journal.pone.0330125 (PMC12364367; doi:10.1371/journal.pone.0330125)
Supplement: Attachment — Submitted filename: Response to Reviewers plos one.docx [file pone.0330125.s004.docx]

| **Reviewer #1 comments** | **Response** |
| --- | --- |
| - In your introduction near line 110 it appears the first sentence of the new paragraph is a repeat of the description/ definition of pulsatile flushing that is mentioned up above. Nothing major it just reads as if you are defining it twice. | Thanks for your comment.   We have revised the sentence in Line 128. |
| - For table 2- I did not see outcome 17 listed as being used in the table. Perhaps it was just not assigned to a paper or you could just remove it as an outcome. | Thank you for your comment.  Outcome 17 was indeed used in Table 2 and assigned to the study by Zhu et al. (2020) on the Page 19. |
| - On section 5.5 on flushing speeds it may be worth considering converting the 4 cm3/sec to ml/sec to have consistency in the labeling of units throughout the manuscript. Again, near sentence at number line 478. | Thank you for your comment.  We have revised the unit from “4cm^3^” to “4mL/s” in the section 5.5 and Line 514. |
| - For table 3 for the last row under the column Gaps- would reword the sentence to read “… patency or prevent complications or damage to the endothelium”. Essentially removing the word damage again at the end. | Thank you for your comment.  We have revised the sentence as seen in Table 3. |
| - Around line number 490- For the data on shear rates- I could not see where ref 67 Dunkley et. al states specific shear rates related to platelet aggregation. And I didn’t see mention of them by Ref 65 Nicholson. Perhaps the reference numbers got shifted a little. | Thank you for your helpful comment.  We have reviewed the references and identified that the original References 65 and 67 did not adequately support the statements regarding physiological and pathological shear rates and their impact on platelet aggregation. We have replaced Reference 65 with Shi et al. (2015), which provides a detailed explanation of shear rate ranges in vein. Reference 67 has been updated to Casa et al. (2016), which reports on the effects of different shear rates on platelet thrombi formation and detachment. Both references have been appropriately cited in the revised text, as seen in Line 791 and Line 796. |
| **Reviewer #2 comments** | **Response** |
| Only point is the possible grammatical error of line 78 and PVC used without the explanation of what it stands for. Was this intended to be PIVC? PVC is later used and explained in line 251. | Thanks for your comment.  It was a typo in Line 78 should have been PIVC (peripheral intravenous catheter). We have corrected this in Line 96. |
